# Pitfalls in Experimental Designs for Characterizing the Transcriptional, Methylational and Copy Number Changes of Oncogenes and Tumor Suppressor Genes

**DOI:** 10.1371/journal.pone.0058163

**Published:** 2013-03-05

**Authors:** Yuannv Zhang, Jiguang Xia, Yujing Zhang, Yao Qin, Da Yang, Lishuang Qi, Wenyuan Zhao, Chenguang Wang, Zheng Guo

**Affiliations:** 1 College of Bioinformatics Science and Technology, Harbin Medical University, Harbin, China; 2 Department of Pathology, University of Texas MD, Anderson Cancer Center, Houston, Texas, United States of America; 3 School of Life Science and Bioinformatics Centre, University of Electronic Science and Technology of China, Chengdu, China; Shanghai Jiao Tong University School of Medicine, China

## Abstract

**Background:**

It is a common practice that researchers collect a set of samples without discriminating the mutants and their wild-type counterparts to characterize the transcriptional, methylational and/or copy number changes of pre-defined candidate oncogenes or tumor suppressor genes (TSGs), although some examples are known that carcinogenic mutants may express and function completely differently from their wild-type counterparts.

**Principal Findings:**

Based on various high-throughput data without mutation information for typical cancer types, we surprisingly found that about half of known oncogenes (or TSGs) pre-defined by mutations were down-regulated (or up-regulated) and hypermethylated (or hypomethylated) in their corresponding cancer types. Therefore, the overall expression and/or methylation changes of genes detected in a set of samples without discriminating the mutants and their wild-type counterparts cannot indicate the carcinogenic roles of the mutants. We also found that about half of known oncogenes were located in deletion regions, whereas all known TSGs were located in deletion regions. Thus, both oncogenes and TSGs may be located in deletion regions and thus deletions can indicate TSGs only if the gene is found to be deleted as a whole. In contrast, amplifications are restricted to oncogenes and thus can be used to support either the dysregulated wild-type gene or its mutant as an oncogene.

**Conclusions:**

We demonstrated that using the transcriptional, methylational and/or copy number changes without mutation information to characterize oncogenes and TSGs, which is a currently still widely adopted strategy, will most often produce misleading results. Our analysis highlights the importance of evaluating expression, methylation and copy number changes together with gene mutation data in the same set of samples in order to determine the distinct roles of the mutants and their wild-type counterparts.

## Introduction

It is well recognized that “activation” of oncogenes and “inactivation” of tumor suppressor genes (TSGs) combine to confer selective advantages on cancer cells [1], [2]. As originally defined, oncogenes and TSGs arise as results of genetic lesions that increase the activity of a proto-oncogene or decrease the activity of a TSG [1], [3]. The genetic lesions contain mutations (including point mutations, intragenic deletions and insertions, chromosomal translocations) and copy number alterations (CNAs). In general, the genetic lesions inducing “activation” of oncogenes or “inactivation” of TSGs can be classified into two types: i) genetic lesions in the coding regions of the genes that can result in hyperactive proteins (for oncogenes) or non-functional proteins (for TSGs) that differ from the normal proteins encoded by the corresponding wild-type genes; ii) genetic lesions in the promoter regions or CNAs of the genes that can enhance or repress the expression of the normal gene products [1], [3].

After identifying genetic lesions in cancer genomes, wet lab experiments and bioinformatics strategies are often used to determine whether the genetic lesions induce the “activation” of oncogenes or the “inactivation” of TSGs. However, almost all researches for characterizing oncogenes and TSGs do not discriminate patients with mutants from patients with their wild-type genes [4]–[15], whereas carcinogenic mutants may express and function completely differently from their wild-type counterparts [16], [17]. For CNAs, the amplification or deletion of the gene is often used to support the gene as a proto-oncogene or a TSG [4]–[7]. Notably, as CNAs can also alter DNA sequences to create mutants [18], this strategy potentially confounds the changes of the mutants and their wild-type counterparts. For the other genetic lesions, researchers usually collect an independent set of samples without gene mutation data to find evidence of transcriptional or methylational changes of this gene in cancer. This widely applied strategy also potentially confounds the changes of the mutants and their wild-type counterparts because the overall change in expression or methylation detected in a cancer versus normal dataset for a gene primarily reflects the change of the wild-type gene in a majority of cancer patients given the low mutation rates for most genes [19]. In addition, current databases of oncogenes and TSGs, such as the Cancer Gene Census database [20] and the tumor suppressor gene database [21], provide no information about the particular mutants that contribute to cancer. Based on such databases, many studies [22], [23] including bioinformatics analyses for training classifiers to predict cancer genes, do not discriminate between wild-type genes and their mutants. This problem may introduce great confusion into our attempts to understand oncogenesis and cancer progression. Importantly, correctly evaluating the possible different carcinogenic roles of a wild-type gene and its mutant is critical for developing personalized treatments as cancer therapies targeting oncogenes and TSG are totally different –– inhibiting oncogene function but restoring TSG function [24], [25].

Therefore, in this study, we systemically evaluated the effectiveness of using the overall expression, methylation and/or copy number changes of genes in cancer tissues without discriminating gene mutation states (including mutants and their wild-type genes) to determine the “activation” of oncogenes or “inactivation” of TSGs. Here, we specified genes previously labeled as oncogenes or TSGs for a particular cancer type without discriminating their mutation states as candidate-oncogenes (C-oncogenes) or candidate-TSGs (C-TSGs) for this cancer type. Based on multiple expression, methylation and copy number profiles for a total of nine cancer types, we firstly showed that, for each cancer type, the directions of overall expression, methylation and copy number changes of a gene without discriminating mutants and their wild-type genes were fixed in properly designed case–control studies. Then, we showed that the overall expression and methylation changes of a gene in cancer provide no evidence for its mutant as an activated oncogene or an inactivated TSG. We also showed that DNA amplification is unique to C-oncogenes and thus amplification of a gene can support either the dysregulated wild-type gene or its mutant as an oncogene, depending on whether the wild-type gene or its mutant is amplified. However, the current practice of using deletion to support a wild-type gene as a TSG [4]–[7] could be valid only if this gene is found to be deleted as a whole because deletions may frequently alter DNA sequences to create mutant oncogenes; otherwise, more evidences are needed to determine whether the partial deletion result in a non-functioning gene product or a functional oncogenic chimera. Finally, a case study of *TP53* shows that *TP53* in different mutation states can be deregulated in different directions in a particular cancer. Our findings highlight the basic importance of evaluating expression, methylation, copy number changes together with gene mutation data in the same set of samples in order to determine the distinct roles of the mutants and their wild-type counterparts.

## Results

### Directions of DE, DM and CNA Genes in Population-level Case–control Studies

Firstly, we evaluated whether the overall change directions of the expression, methylation and copy number of a gene for a particular cancer are fixed in population-level case–control studies using randomly sampled cancer tissues and normal controls without discriminating gene mutation states. Using the significance analysis of microarrays (SAM) method [26], with 5% false discovery rate (FDR) control, we selected two lists of differentially expressed genes (DE genes) from the two datasets for each of the eight cancer types that had two expression profiles available (see *Materials* and *methods*). Only 67% of the DE genes shared by the two lists for prostate cancer had consistent up- or down-regulation status (Table 1), indicating that the reliability of these DE genes was relatively low. For each of the other seven cancer types, above 90% of the DE genes shared by the two lists of DE genes had consistent up- or down-regulation status (Table 1), which was unlikely to occur by chance (Bernoulli *p*<2.20×10^−16^). Additionally, we calculated the directional consistency of the union of DE genes identified in the two datasets for each cancer type. The results showed that averagely 87% of the DE genes identified in at least one dataset had consistent up- or down-regulation status across the two datasets for each of the seven cancer types analyzed in this study (Table S1), which was unlikely to occur by chance (Bernoulli p<2.20×10−16). Notably, as demonstrated in our previous work [27], many gene truly differentially expressed in a disease may not always show differential expression signals in a particular dataset for this disease especially when the sample size is small and/or the measurement noise is high. If a “true” DE gene is not identified as significantly changed in a dataset, it is likely that its change direction in this dataset may fluctuate randomly. Thus, it would be more reasonable to evaluate the reliability of DE genes identified in only one dataset for a cancer type using the change directions of those genes showing at least marginally significant changes (e.g., p<0.1) in another dataset. The comparison showed that averagely 96% of such DE genes had consistent up- or down-regulation status across the two datasets for each of the seven cancer types (Table S2, Bernoulli p<2.20×10−16). Furthermore, we proved the reliability of the non-overlapping DE genes identified from the two datasets for each of the seven cancer types using the DE genes in another independent dataset for this cancer (Table S3). The results indicated that the DE genes identified in each dataset for each of the seven cancer types can reliably capture a portion of the total deregulated genes for that cancer. Thus, in the following analyses, we focused on analyzing DE genes of these seven cancer types and used DE genes identified in at least one dataset for each cancer type but ignored those with opposite changes in regulation.

**Table 1:** Directional agreement of DE, DM and CNA genes.

Cancer types	DE geneswith 5% FDR	DM genes with 5% FDR	CNA genes with 5% FDR	CNA genes with 1% FDR
Lung	99.86% (4253)	99.64% (2771)	73.31% (1881)	90.78% (781)
Gastric	99.88% (1675)	99.46% (2573)	NA	NA
Renal	98.11% (4549)	99.76% (4505)	NA	NA
Prostate	66.59% (458)	NA	100% (100)	100% (7)
Breast	95.93% (6947)	NA	68.02% (544)	100% (365)
Pancreatic	98.41% (9096)	NA	NA	NA
Ovarian	98.95% (5427)	NA	NA	NA
Colorectal	92.52% (7802)	99.68% (2832)	99.76% (413)	100% (410)
Brain	NA	NA	100% (188)	100% (113)

Note: For the two lists of DE, DM or CNA genes detected from two datasets for each cancer, the directional agreement rates were calculated as the number of DE, DM or CNA genes with consistent directions across the two datasets divided by the number of all DE, DM or CNA genes commonly detected in both datasets (in parenthesis); NA, not available.

Using the Student's t test, with 5% FDR control, we selected two lists of differentially methylated genes (DM genes) from the two datasets for each of the four cancer types that had two methylation profiles available (see *Materials* and *methods*). We observed very consistent directionality in methylation changes across datasets for each of the four cancer types (Table 1). Additionally, we found that averagely 98% of the DM genes identified in at least one dataset had consistent hypomethylation or hypermethylation status across the two datasets for each of the four cancer types analyzed in this study (Table S1, Bernoulli p<2.20×10−16). Furthermore, we proved the reliability of the non-overlapping DM genes identified from the two datasets for each of the four cancer types using the change directions of those genes showing marginally significant changes (p<0.1) in these two datasets or DM genes in another independent dataset for this cancer (Table S2, Table S3). The results indicated that the DM genes identified in each dataset for the four cancer types can reliably capture a portion of the total genes with methylation changes for that cancer. Thus, in the following analyses, we focused on analyzing DM genes of these four cancer types and used DM genes identified in at least one dataset for each cancer type but ignored those with opposite methylation changes.

Using the GISTIC (Genomic Identification of Significant Targets in Cancer) algorithm, with 5% FDR control, we selected CNAs for each of the five cancer types that had two copy number profiles available (see *Materials* and *methods*). For each of the colorectal, prostate and brain cancer types, more than 99% of the CNA genes detected in both datasets were consistent in the amplification or deletion status, which was unlikely to occur by chance (Bernoulli *p*<2.20×10^−16^). Additionally, for each of the three cancer types, if the non-overlapping CNA genes were also found to be located in CNA regions in the third dataset, then averagely 93% of them were consistent in the amplification or deletion status in the third dataset (Table S3). The concordance rates were only 73% for lung and 68% for breast cancers but increased to 100% and 91%, respectively, when a stricter FDR control of 1% was used (Bernoulli *p*<2.20×10^−16^; Table 1). Thus, the CNAs selected with 1% FDR were used for these two cancer types in the following analyses. With 1% FDR, for the non-overlapping CNA genes for each of the two cancer types that were also found to be located in CNA regions in the third dataset for that cancer type, then averagely 93% of them were consistent in the amplification or deletion status in the third dataset (Table S3). Also, for each of the five cancer types, we used CNA genes identified in at least one dataset but ignored those with inconsistent changes in the two datasets.

The strong concordance in the directionality of a particular type of molecular changes (expression, methylation or copy number changes) across different datasets for a cancer type indicates that the directions of the changes are fixed in properly designed population-level cancer versus normal studies. Therefore, in the following text, we focused on C-oncogenes and C-TSGs with changes in expression, methylation and copy number in the above-mentioned seven, four and five cancer types, respectively.

### Candidate-oncogenes

**Figure 1:**
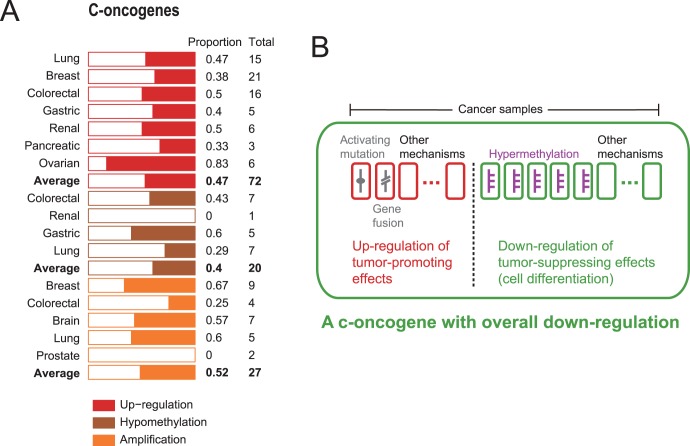
The expression, methylation and copy number changes of C-oncogenes and the related mechanisms. **A**, Numbers of C-oncogenes with expression, methylation and copy number changes in the corresponding cancer types. A full list of these C-oncogenes is given in Table S4. **B**, The mechanisms underlying the overall down-regulation of C-oncogenes in cancer patient population.

Then, we evaluated whether C-oncogenes tend to be up-regulated, hypomethylated or amplified in population-level cancer versus normal studies for their corresponding cancer types. In only one of the seven cancer types for analyzing expression changes of C-oncogenes, more than 50% of the corresponding DE C-oncogenes were up-regulated (Figure 1A; Table S4). The average frequency of down-regulation events of C-oncogenes in their corresponding cancer types was 53% (Figure 1A; Table S4). Also, more than 50% of the DM C-oncogenes were hypermethylated in three of the four cancer types for analyzing methylation changes of C-oncogenes. The average frequency of hypermethylation events of C-oncogenes in their corresponding cancer types was 60% (Figure 1A; Table S4). Thus, C-oncogenes do not tend to be up-regulated or hypomethylated in their corresponding cancer types.

The reason for the above results could be that the overall down-regulation or hypermethylation of a C-oncogene in a cancer versus normal study for a particular cancer type tends to be determined from a majority of cancer samples that contain the wild-type gene rather than its mutant based on which the gene is labeled as “oncogene”, whereas a wild type gene may play a different carcinogenic role from its mutant. For example, *FGFR2* was identified as an oncogene for the lung cancer based on the observation that the W290G mutant can promote lung cancer by stimulating growth factor signaling [24]. However, we found that *FGFR2*, which participates in cell differentiation [28], was down-regulated and hypermethylated in lung cancer. Given the low mutation rate of *FGFR2* in the lung cancer [19], down-regulation of the wild-type *FGFR2* may compromise cell differentiation and promote cancer in most patients of the lung cancer. Notably, we found that many C-oncogenes down-regulated in their corresponding cancer types were involved in cell differentiation whose arrest contributes to carcinogenesis [29] (Table S5). Similarly, many hypermethylated C-oncogenes were also involved in cell differentiation [30] (Table S5). Thus, down-regulation or hypermethylation of many wild-type C-oncogenes normally involved in cell differentiation may be predominant contributors to cancer (Figure 1B).

In the five cancer types for analyzing copy number changes of C-oncogenes, we found 14 amplification events involving 12 C-oncogenes and 13 deletion events involving 11 C-oncogenes in their corresponding cancer types (Table S4). Thus, C-oncogenes do not tend to be amplified in their corresponding cancers (Bernoulli *p = *0.5; Figure 1A). The result showed that about half of the C-oncogenes were located in deletion regions, indicating that deleted regions might be associated with DNA sequence alterations such like gene fusion (Figure 1B) that create oncogenic mutants. For the 11 C-oncogenes with deletions in their corresponding cancer types, four genes (*ERG*, *TMPRSS2*, *ROS1* and *GOPC*) can create oncogenic fusion genes (*ERG-TMPRSS2* and *ROS1-GOPC*), and deletions in another four (*CD74*, *BCL2*, *TRIM33* and *ETV6*) can lead to fusion oncogenes by translocation, as shown in the Mitelman Database of Chromosome Aberrations in Cancer (http://cgap.nci.nih.gov/Chromosomes/Mitelman) and previous studies [20], [31]. Notably, four C-oncogenes (*NOTH1*, *FGFR2* and *MYC* for the breast cancer and *FGFR3* for the colorectal cancer) were found to be amplified but down-regulated in their corresponding cancer types. The amplifications of these four genes may be associated with DNA sequence alterations to create oncogenic mutants [20], [24], and their overall down-regulation may be typical of their wild-type counterparts that normally participate in cell differentiation and the arrest of which can promote carcinogenesis (Figure 1B) [29].

### Candidate-TSGs

**Figure 2:**
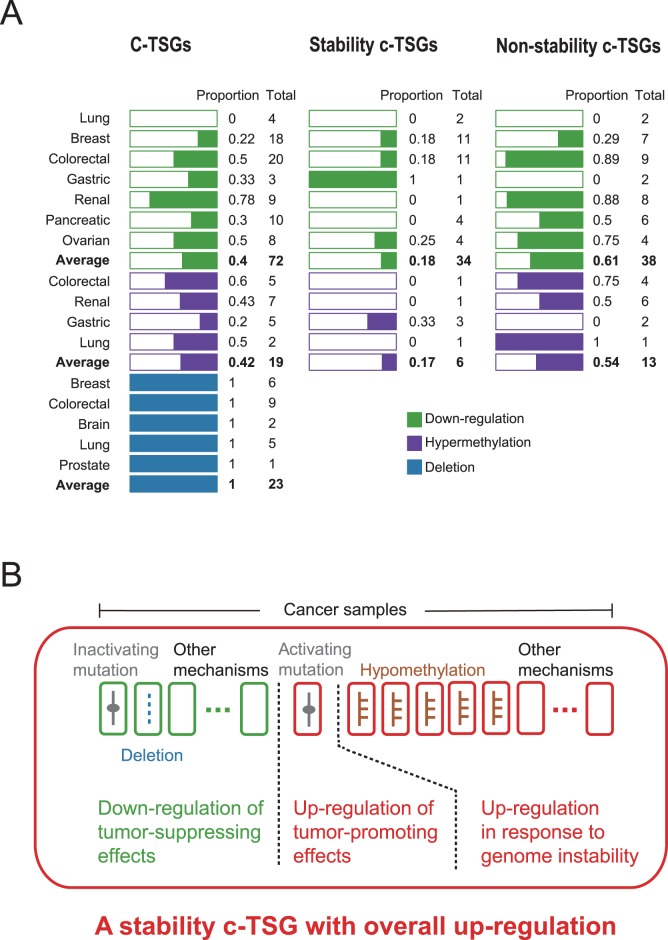
The expression, methylation and copy number changes of C-TSGs and the related mechanisms. **A**, Numbers of C-TSGs, including stability C-TSGs and non-stability C-TSGs, with expression, methylation and copy number changes in the corresponding cancer types. A full list of these C-TSGs is given in Table S4. **B**, The mechanisms underlying the overall up-regulation of stability C-TSGs in cancer patient population.

Similarly, we tested whether C-TSGs tend to be down-regulated, hypermethylated or deleted in population-level cancer versus normal studies for their corresponding cancer types. In six of the seven cancer types for analyzing expression changes of C-TSGs, no more than 50% of DE C-TSGs were down-regulated. The average frequency of down-regulation events of C-TSGs in their corresponding cancer types was only 40% (Figure 2A; Table S4). Also, the average frequency of hypermethylation events of C-TSGs in their corresponding cancer types was only 42% (Figure 2A; Table S4). These results showed that C-TSGs do not tend to be down-regulated or hypermethylated in their corresponding cancers.

Another type of cancer genes, “stability TSGs”, are often classified as TSGs because their inactivation could produce an oncogenic effect but their roles in carcinogenesis are completely different from those of other TSGs [2]: the loss-of-function mutations or the deletion of a stability TSG promote inactivation of other tumor-suppressor genes as well as activation of oncogenes. Therefore, we also analyzed “stability TSGs” and “non-stability TSGs” separately. The result showed that the average frequency of down-regulation events of non-stability C-TSGs in their corresponding cancer types was 61% (Bernoulli *p = *0.12; Figure 2A; Table S4) and the average frequency of hypermethylation events was 54% (Bernoulli *p = *0.5; Figure 2A; Table S4). These results did not suggest that non-stability C-TSGs tend to be down-regulated and hypermethylated in their corresponding cancer types. In contrast, the average frequencies of up-regulation events and hypomethylation events of stability C-TSGs in their corresponding cancer types were 82% (Bernoulli *p* = 9.76×10^−5^) and 83% (Bernoulli *p = *0.11; Figure 2A; Table S4), respectively. These results indicated that stability C-TSGs tend to be up-regulated and hypomethylated in their corresponding cancer types, possibly in response to genomic instability (Figure 2B). As for the up-regulated or hypomethylated non-stability C-TSGs, some of them, such as *SMARCA4* in the lung cancer, may be unknown stability C-TSGs [28]. The up-regulation or hypomethylation of a C-TSG could also reflect the fact that the wild-type C-TSG can assist a mutant oncogene in promoting carcinogenesis [32] or that it can be activated by mutations that make it carcinogenic (Figure 2B) [16], [17].

In the five cancer types for analyzing copy number changes of C-TSGs, we observed no amplification events and 23 deletion events involving 16 C-TSGs in their corresponding cancer types, which was unlikely to occur by chance (Bernoulli *p* = 1.19×10^−7^; Figure 2A; Table S4). Notably, four (*TP53* for the lung cancer, *ATM* for the colorectal cancer, *CDKN2A-p16(INK4a)* and *BRCA1* for the breast cancer) of the 16 deleted C-TSGs were significantly up-regulated in their corresponding cancer types, and all four play roles in maintaining genome stability [28]. The up-regulation trend for these stability C-TSGs mainly reflects changes of their wild-type genes, rather than of the deleted genes themselves (Figure 2B). The up-regulation of the wild-type versions of these four stability C-TSGs indicates that they may respond to genome instability in most patients.

### Distinct Deregulation Pattern of Cancer Genes in Different Mutation States: a Case Study of TP53

**Figure 3:**
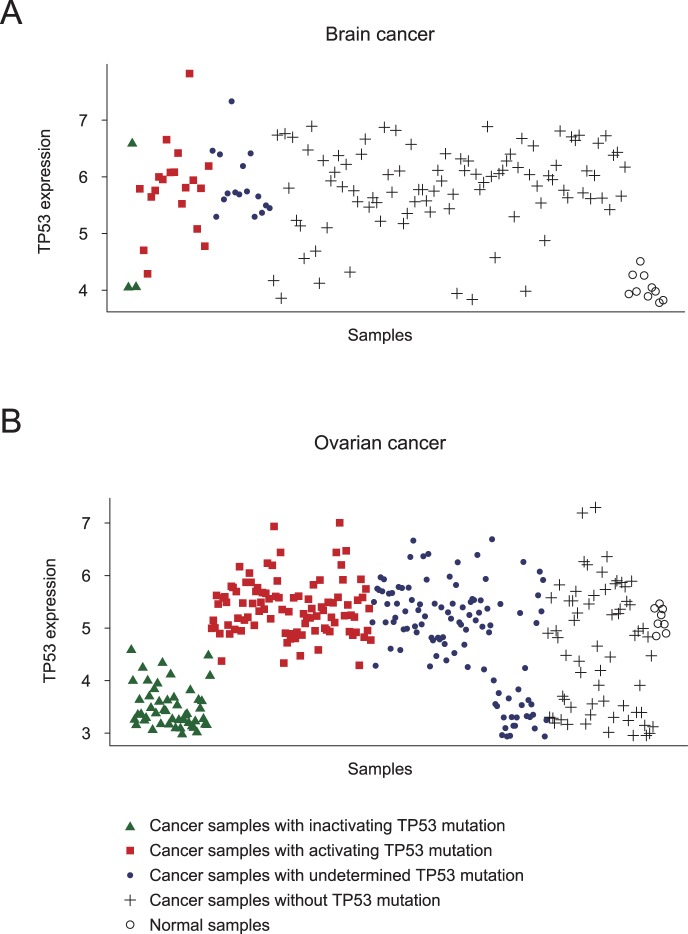
*TP53* in different mutation states can be deregulated in different directions in a cancer type. **A**, In the ovarian cancer, *TP53* with activating mutations was up-regulated, whereas both inactivated *TP53* and wild-type *TP53* were down-regulated; **B**, In the brain cancer, *TP53* with the activating mutations and the wild-type *TP53* were up-regulated, whereas *TP53* with inactivating mutations were not found to be significantly changed possibly due to the small sample size.

The above results suggest the importance of discriminating genes in different mutation states for correctly characterizing the “activation” of oncogenes or the “inactivation” of TSGs. To illustrate this view specifically, we analyzed a typical case, C-TSG *TP53*, using a dataset containing both gene sequence and expression data from 319 samples of ovarian cancer and eight normal controls. We found 254 mutations but no copy number alterations in TP53 in ovarian cancer. Mutations in *TP53* were divided into three types according to the International Agency for Research on Cancer (IARC) TP53 database [33]: activating, inactivating (nonsense, frame shift) and undetermined mutations. As shown in Figure 3A, the expression levels of *TP53* with activating mutations in the 98 ovarian cancer samples were significantly higher than the expression levels of *TP53* in the eight normal controls (*p* = 0.044, two tailed t-test). Yet, the expression levels of both *TP53* with inactivating mutations in the 49 ovarian cancer samples (*p* = 2.61×10^−11^, two tailed t-test) and wild-type *TP53* in the 65 ovarian cancer samples (*p* = 0.001, two tailed t-test) were significantly lower than the expression levels of *TP53* in the normal controls. For brain cancer, we found 31 mutations but no copy number alterations. Similar analysis for brain cancer showed that activated *TP53* was up-regulated in the cancer samples versus the normal controls (*p* = 3.40×10^−9^, two tailed t-test), whereas wild-type *TP53* were down-regulated in the cancer samples versus the normal controls (*p* = 1.49×10^−18^, two tailed t-test; Figure 3B). The expression levels of *TP53* with inactivating mutations in the three brain cancer samples were not significantly different from the expression levels of *TP53* in the normal controls (*p* = 0.421, two tailed t-test) possibly due to the small sample size. The above results suggest that *TP53* in different mutation states can be deregulated in different directions in a particular cancer type.

## Discussion

Based on various high-throughput data without mutation information for typical cancer types, we found that about half of known C-oncogenes (or C-TSGs) pre-defined by mutations were down-regulated (or up-regulated) and hypermethylated (or hypomethylated) in their corresponding cancer types. The comparison with the 50% random chance up-regulation (or down-regulation) indicated that the C-oncogenes (or C-TSGs) show random up- or down-regulation in cancer versus normal control. In most cancer types we analyzed, the up-regulated (or down-regulated) frequencies of C-oncogenes (or C -TSGs) were even lower than the expected frequencies of up-regulated (or down-regulated) DE genes. This result suggested that C-oncogenes are not more likely to be up-regulated (or down-regulated) than the other DE genes. Similarly, the hypomethylated (or hypermethylated) frequencies of C-oncogenes (or C-TSGs) were even lower than the expected frequencies of hypomethylated (or hypermethylated) DM genes (Table S6). Our results suggest that the population-level expression and methylation changes of a gene without considering its mutation states provides no discriminating information about the activation or inactivation of the carcinogenic mutant in cancer as they mainly reflect changes that occur in the wild-type counterpart. As gene activity is regulated at both transcriptional and translational levels, it is possible that some of the observed inconsistency may result from translational or post-translational regulation. On the other hand, even pathways that operate primarily through translational or posttranslational mechanism leave recognizable gene expression signatures [34]–[36], it is largely reasonable to evaluate the transcriptional changes of oncogenes and tumor suppressor genes. Also, we found that only half of the C-oncogenes were located in amplification regions, while the amplified frequencies of C-oncogenes were still higher than the expected frequencies of amplified CNA genes in most caner types we analyzed (Table S6). However, all C-TSGs were located in deletion regions and the deleted frequencies of C-TSGs were higher than the expected frequencies of deleted CNA genes in all caner types we analyzed (Table S6). Our analyses indicate that the amplification of a gene can support either the dysregulated wild-type gene or its mutant as an oncogene, depending on whether the wild-type gene or its mutant is amplified. However, the deletion could be valid for supporting a wild-type gene as a TSG only if we could find other evidence that the whole gene is deleted as deletions may frequently alter the DNA sequences to create oncogenic mutants; otherwise, more evidences are needed to determine whether the partial deletion result in a non-functioning gene product or a functional oncogenic chimera. Taken together, our results clearly show that the currently widely applied experimental strategy which uses the overall expression, methylation and/or copy number changes of genes in cancer tissues without discriminating gene mutation states to determine the “activation” of oncogenes or “inactivation” of TSGs is likely to produce misleading results. It is worth noting that many studies are also hindered by two other problems: a lack of statistical significance owing to insufficient small sample sizes [11], [37], and bias introduced by considering only samples for which the expression, methylation and/or copy number changes are concordant with the supposed roles of the genes as oncogenes or TSGs [9], [11].

In order to distinguish the changes of a carcinogenic mutant and its wild-type counterpart, the expression, methylation and copy number changes of a candidate cancer gene in cancer should be studied in the same set of samples used for mutational analysis. Currently, The Cancer Genome Atlas (TCGA) project [38] and the International Cancer Genome Consortium project [39] are generating data of mutation and other molecular changes from the same sets of samples, which provide basis for discriminating molecular changes of a gene in different mutation states. However, the availability of such multi-dimensional data is still very limited [38], [40]–[44], and the lack of expression data from healthy controls is particularly problematic (for example, some studies did not include any [38], [40] or had only two normal samples [41], [42]). To tackle the problem of incomplete data, some researchers have used gene expression levels from cancer samples without mutations as the baseline for measuring the deregulation of mutated genes in cancer [38], [40]. This is problematic because wild-type C-oncogenes and C-TSGs also tend to be differentially expressed in cancer. Thus, a sufficient number of healthy controls are required to determine the true baseline expression levels of genes in healthy tissues. Another problem is that the low mutation rates of most cancer genes limit the statistical power of determining the changes of the mutants. It would be a cost-effective approach to use paired healthy tissue to determine the baseline expression of genes for each individual, which also allows us to examine whether a wild-type or its mutant is dysregulated in the same direction in carcinogenesis for different patients. This is an interesting question that merits future study.

Our analysis suggests that many down-regulated or hypermethylated wild-type C-oncogenes in cancers are normally involved in cell differentiation and that their down-regulation may repress differentiation, resulting in continued proliferation of cells and a failure to die [45] which may confer self-renewing properties on cancer stem cells during carcinogenesis [46]. Our results also show that wild-type stability C-TSGs tend to be significantly up-regulated and hypomethylated in cancer, unlike other C-TSGs, supporting the idea that stability C-TSGs should be regarded as a separate class of cancer genes. In general, a carcinogenic mutant and its wild-type counterpart may express and function differently and their respective roles should be determined separately. The failure to consider this difference may account for the poor performance of current classifiers to discriminate C-oncogenes from C-TSGs [22], [23].

Finally, we highlight that correctly discriminating the carcinogenic roles of a mutant and its wild-type counterpart is important for effective personalized treatments as cancer therapies targeting oncogenes and TSGs aim at inhibiting oncogene function and restoring TSG function, respectively [24], [25]. The effectiveness of a therapy is often tested on cells harboring a mutated oncogene, but in many cases, the drugs do not specifically target the mutated gene [24]. Our results show that some wild-type C-oncogenes might play roles in suppressing carcinogenesis. In such cases, drugs inhibiting the wild-type C-oncogene could cause adverse effects in a majority of patients who already possess a down-regulated wild-type C-oncogene. Therefore, the functional effects of a drug on patients with wild-type C-oncogenes should be evaluated before the drug is used in the clinic. Similarly, we should take care when considering cancer gene therapies that restore TSG function because the wild-type C-TSG may assist a mutant oncogene in promoting carcinogenesis [32] or may be vulnerable to activating mutations [16], [25]. Thus, discriminating the roles of a wild-type gene and its mutants is important for developing personalized treatments, which is becoming practical as genome-scale sequencing and microarray technologies are becoming more efficient and cost-effective.

## Materials and Methods

### C-oncogenes, C-TSGs and Stability C-TSGs

C-oncogenes and C-TSGs for lung, gastric, renal, prostate, breast, pancreatic, ovarian, colorectal and brain cancers were obtained from F-Census [47], which is a collection of cancer genes from various data source including the Cancer Gene Census dataset [20] and the Tumor Suppressor Gene database [21]. In the Cancer Gene Census database, C-oncogenes and C-TSGs are labeled according to whether mutations are dominant or recessive [48]. In the Tumor Suppressor Gene database, C-TSGs were collected by text mining from the National Center for Biotechnology Information and other credible data sources [21]. For all the nine cancer types analyzed in this paper, no gene is labeled as both C-oncogene and C-TSG. The C-oncogenes and C-TSGs analyzed in this work are shown in Table S7.

We divided C-TSGs into stability and non-stability C-TSGs by extracting the stability C-TSGs in Table 1 of [2]. In addition, we also considered C-TSGs assigned to the Gene Ontology terms “DNA repair”, “cell cycle checkpoint” and “response to DNA damage stimulus” as stability C-TSGs [28]. The Gene Ontology annotation data were downloaded on April 29, 2011 [28].

### Expression, Methylation and Copy Number Profiles

From the Gene Expression Omnibus [49], TCGA [38] and Tumorscape [18] databases, we collected three types of molecular profiles (expression, methylation and copy number changes) for different cancers according to the following criteria: for each type of molecular changes of a particular cancer type, there had to be at least two profiles available for evaluating the concordance of this type of molecular changes across different studies [50]; and each profile had to include at least 10 cancer samples and 10 healthy controls, respectively. When more than two profiles for a particular type of molecular changes were available for a cancer type, the two profiles with the largest sample sizes were adopted. According to the above criteria, we obtained two expression profiles for each of eight cancer types, two methylation profiles for each of four cancer types and two copy number profiles for each of five cancer types. No samples were shared by any two profiles of each cancer type. As for the third profile used to validate the reliability of non-overlapping differential genes discovered from the above two profiles for each type of molecular changes of a particular cancer type, the profiles with a smaller sample size were also adopted if no profile was available according to the above criteria. All of the datasets analyzed in this work are described in Table S8. Notably, because no copy number data from normal samples were available for breast cancer and prostate cancer in these databases, we used the 48 healthy samples from the Affymetrix website (http://www.affymetrix.com).

For the case study of TP53, we used two datasets containing both gene sequence and expression profiles for the brain and ovarian cancers, respectively, downloaded from the TCGA database [38].

### Selection of DE, DM Genes and CNAs

The raw gene expression data were normalized by the robust multi-array average (RMA) algorithm. We used background-adjusted PM probe intensities [51] and mapped the probe sets to Entrez genes based on the SOURCE database (downloaded in July, 2010) [52]. If multiple probes were mapped to a single gene, we adopted the average of the probe intensities as the expression value of this gene. DE genes in cancer samples versus normal controls were selected by the SAM method (samr_1.25 R package) [26].

We used the level 2 TCGA data, which provides U (un-methylated) and M (methylated) values for each probe. The beta-value of a probe was calculated as M/(U+M+100). The probe IDs were mapped to gene IDs by the annotation table for each platform [53]. DM genes in cancer samples versus normal controls were selected based on the Student's t test [53]. The P-values were adjusted by the Benjamini and Hochberg method for multiple-testing correction [54].

The probe-level signal intensities of the DNA copy number data were normalized using invariant set normalization [55], and the SNP-level signal intensities were obtained using a model-based method [56]. Significant CNAs were determined using the GISTIC method [57]. SNP, gene and cytogenetic band locations were based on the hg17 (May 2004) genome build [58].

### Statistical Analysis for Consistent Directionality of a Type of Molecular Change

If there were *N* DE genes that were commonly detected in two independent profiles for a cancer, then the significance level (the random chance) of observing at least *m* DE genes with consistent up- or down-regulation directions across the two profiles was calculated by using the binomial distribution model as follows:
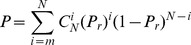
where *P*_r_ ( = 0.5) is the probability of observing by random chance that a DE gene shared by the two lists is detected to be both up-regulated or both down-regulated in the corresponding two profiles.

Similar statistical analysis was done for consistent directionality of methylation changes (hypermethylation or hypomethylation) of two lists of DM genes and for consistent directionality of CNAs (amplification or deletion) of two lists of CNA genes.

## Supporting Information

Table S1 Directional agreement of the union of DE and DM genes across two datasets for each cancer type.
(XLS)

Table S2 Directional agreement of the DE and DM genes, which were significantly changed in one dataset and at least marginally significantly changed in another dataset, across two datasets for each cancer type.
(XLS)

Table S3 Directional agreement of non-overlapping DE, DM and CNA genes in the third dataset for each cancer type.
(XLS)

Table S4 All cancer genes with differential expression, methylation or copy number changes in their corresponding cancer types.
(XLS)

Table S5 Down-regulated or hypermethylated c-oncogenes involved in cell differentiation.
(XLS)

Table S6 The comparison between the altered frequencies of C-oncogenes (C-TSGs) and the expected altered frequencies.
(XLS)

Table S7 C-oncogenes, c-TSGs and stability c-TSGs for nine cancer types.
(XLS)

Table S8 Expression, methylation and copy number datasets used in this study.
(XLS)
